# Acceptability, Perceived Feasibility and Perceived Impact of an Acceptance and Commitment Therapy-Informed Emotion Regulation Program to Assist Parents in Supporting Their Children’s Physical Activity: A Qualitative Proof-of-Concept Study

**DOI:** 10.3390/bs16071172

**Published:** 2026-07-11

**Authors:** Alfred S. Y. Lee, Alyssa T. K. Manankil-Lakusta, Ryan E. Rhodes

**Affiliations:** Behavioural Medicine Laboratory, Faculty of Education, School of Exercise Science, Physical and Health Education, University of Victoria, P.O. Box 3010 STN CSC, Victoria, BC V8W 3N4, Canada; manankil@uvic.ca (A.T.K.M.-L.); rhodes@uvic.ca (R.E.R.)

**Keywords:** MVPA, online workshops, parental support, emotional regulation, acceptance and commitment therapy

## Abstract

Insufficient physical activity (PA) among children remains a public health concern, and parental support is a consistent correlate of child PA. Existing family-based PA interventions have shown modest effects, suggesting a need to address emotional barriers that interfere with parents’ support follow-through. This Phase IIa proof-of-concept study examined the acceptability and perceived feasibility of a remotely delivered Acceptance and Commitment Therapy-informed emotion regulation program for parents of children not meeting PA guidelines, and explored perceived changes in parental support. Twenty-one Canadian parents of children aged 6 to 12 years began three 20 to 30 min parent-only online workshops delivered over three weeks; 15 completed all workshops and 12 completed a semi-structured exit interview. Cross-case thematic analysis generated three themes. Parents described the program as acceptable, accessible, and supportive; reported that strategies were feasible to apply and helped them support PA in more flexible, less perfectionistic, and more values-consistent ways; and identified refinements, including stronger support for sustained engagement, greater interactivity, and child inclusion where appropriate. Findings suggest preliminary promise and support further optimization and mixed-method testing of measurable changes in parental support and child or family PA.

## 1. Introduction

Insufficient physical activity (PA) among children and adolescents remains a major global public health concern (Guthold et al., 2020). Across international surveillance systems, low levels of PA are consistently observed among children and adolescents worldwide, and, at the global level, 81% of adolescents aged 11 to 17 years are insufficiently active, indicating that this problem is widespread during the developmental years (Aubert et al., 2021; Guthold et al., 2020). In Canada, the concern is similarly pronounced, with only 39% of children and youth aged 5 to 17 years meeting the recommendation of at least 60 min of moderate- to vigorous-intensity physical activity (MVPA) per day (ParticipACTION, 2024). Regular MVPA during childhood and adolescence is associated with better cardiorespiratory and muscular fitness, bone and cardiometabolic health, cognitive and academic functioning, mental health, and healthier adiposity profiles (Chaput et al., 2020). Given these benefits and the persistently low proportion of children meeting PA guidelines, effective PA promotion remains a priority. Family-based approaches are especially relevant because children’s movement behaviours are shaped within everyday family routines, resources, and support practices, and family-based intervention and qualitative synthesis evidence highlights parents as central agents in creating opportunities for child PA (Brown et al., 2016; Rhodes et al., 2020b). Accordingly, efforts to address persistent childhood inactivity should attend to modifiable influences within the family context, particularly the ways parents encourage, facilitate, model, and co-participate in their children’s PA (Pyper et al., 2016; Yao & Rhodes, 2015).

Parental support has been identified as one of the most important family-level influences on children’s PA because the family is a key context in which children’s movement behaviours are encouraged, facilitated, modelled, and enacted (Edwardson & Gorely, 2010; Rhodes et al., 2019b; Trost & Loprinzi, 2011). Within the child PA literature, parental support is most commonly operationalized as behaviours such as encouragement, logistical support or facilitation (e.g., transportation, enrolment, access to opportunities), and co-participation or role modelling (Pyper et al., 2016; Rhodes et al., 2020b). Reviews consistently identify parental support as positively associated with child PA (Edwardson & Gorely, 2010; Trost & Loprinzi, 2011), and a meta-analysis found that parental support showed a moderate association with child PA, *r* = 0.38 (95% CI 0.30–0.46), whereas parental modelling showed a weaker but still positive association, *r* = 0.16 (95% CI 0.09–0.24; Yao & Rhodes, 2015).

Despite this observational evidence, family-based PA interventions focused on parental support of child PA have produced only modest overall effects on child PA, with Brown et al. (2016) reporting a sensitivity-adjusted effect of standardized mean difference (SMD) = 0.29 (95% CI 0.14–0.45), and prior family-based trials similarly suggest that gains produced through education, planning, and coping strategies can attenuate over time (Rhodes et al., 2010, 2019a, 2021). This pattern suggests that the central challenge is not simply helping parents form supportive intentions, but helping them enact and maintain support repeatedly in the context of everyday family life (Lithopoulos et al., 2020).

Related qualitative work shows that providing consistent support can be derailed by lack of time, bad weather, incompatible parent–child preferences, parental health limitations, cost, safety concerns, screen-related issues, and negative affective experiences associated with support itself (Brennan et al., 2025). These findings suggest that improving parental support may require more than education and planning alone; it may also require helping parents respond more effectively to stress, fatigue, frustration, guilt, and other difficult internal experiences that can interrupt supportive action in daily family life.

Emotion regulation provides a useful lens for this problem because it refers to how individuals influence, manage, and respond to emotional experiences in ways that support well-being and goal-directed action (Dixon-Gordon et al., 2015; Gross, 2015). In parenting, emotion regulation is particularly relevant because parents must manage their own emotional responses while continuing to provide support, guidance, and structure in demanding family situations (Rutherford et al., 2015; Zimmer-Gembeck et al., 2022). Broader parenting research suggests that parent emotion regulation is associated with parenting behaviour and child adjustment (Zimmer-Gembeck et al., 2022), and reviews of emotion regulation in parenthood emphasize that parents must not only maintain their own regulated state, but also support children’s developing regulation (Rutherford et al., 2015). In the present context, this is important because many parents already value supporting their child’s PA, yet still have to follow through in the presence of stress, fatigue, guilt, frustration, and other difficult internal experiences that can disrupt supportive behaviour in daily family life (Stevens et al., 2020). Thus, the challenge may not simply be helping parents know what to do but helping them respond to these internal experiences in ways that preserve supportive action when it matters most.

Emotion regulation is not a single technique but a broad set of strategies. Contemporary models distinguish multiple strategy families, including antecedent-focused approaches such as situation selection (choosing or modifying exposure to emotion-eliciting situations), attentional deployment (shifting attention within a situation), and cognitive change or reappraisal (altering how a situation is interpreted), as well as response-focused approaches such as suppression (inhibiting or changing emotional expression after an emotion has been generated; Gross, 1998, 2015). Importantly, this literature also emphasizes that no single strategy is universally optimal; instead, effective emotion regulation depends on context and flexible responding to situational demands (Gross, 2015). In the PA domain, affective and emotion regulation processes are increasingly recognized as relevant to whether people initiate, enact, and sustain activity, and recent work suggests that emotion regulation intervention approaches are now emerging as a distinct line of PA research (Rhodes et al., 2025; Stevens et al., 2020). Candidate approaches in this broader space therefore include reappraisal-oriented cognitive approaches as well as acceptance-based approaches that seek to change how people respond to aversive internal experiences during behaviour enactment. For the present parental support context, an acceptance-based approach is especially relevant because the problem is not only the presence of difficult emotions, but whether parents can continue acting supportively when those emotions arise. Acceptance and Commitment Therapy (ACT) is therefore a promising fit for parental support of child PA.

ACT is a contemporary behavioural approach that aims to increase psychological flexibility, or the capacity to pursue values-consistent actions in the presence of difficult thoughts and emotions rather than being dominated by them (Hayes et al., 2006, 1999). ACT is commonly described in terms of six core processes: (1) acceptance, or making space for difficult emotions; (2) cognitive defusion, or stepping back from unhelpful thoughts; (3) present-moment awareness, or bringing attention to what is happening here and now; (4) values, or identifying what truly matters; (5) committed action, or taking meaningful steps in line with those values; and (6) self-as-context, or viewing oneself as more than any passing thought, feeling, or setback (Hayes et al., 2006). These processes are intended to help people respond to internal experiences with greater openness and awareness while continuing to move in valued directions (Hayes et al., 2006). In this way, ACT is relevant to parental support for child PA because it shifts the focus from trying to eliminate distress to helping parents continue acting in line with valued parenting directions even on difficult days (Bassett-Gunter et al., 2017).

Evidence from adjacent studies is encouraging. At the broader health-behaviour level, ACT has been proposed as a contextually driven approach for supporting behaviour change by helping individuals act in line with values while responding flexibly to difficult thoughts, emotions, and situational demands (Zhang et al., 2018). In the PA domain, a meta-analysis (Pears & Sutton, 2021) found that ACT-based PA interventions produced a small-to-moderate positive effect on PA (SMD = 0.32, 95% CI 0.07–0.57). More recently, Rhodes et al. (2025), in a broader contemporary review of emotion regulation interventions for PA, reported that ACT-based interventions also showed a positive effect on PA in subgroup analyses (g = 0.25, 95% CI 0.04–0.46), although the authors noted that estimates across this literature remain influenced by preliminary-phase studies. A recent ACT-informed web-based PA feasibility trial (Grant et al., 2025) reported improvements in MVPA, emotion regulation, behavioural regulation, affective attitude, identity, mindfulness, and valued living in adults. Parallel work in parent populations also suggests that ACT can improve psychological flexibility and reduce distress, with preliminary evidence for benefits to parenting behaviour, although most of this work has been conducted in chronic or neurodevelopmental contexts rather than in interventions targeting support for children’s PA specifically (Byrne et al., 2021; Feldman et al., 2023; Jin et al., 2021).

Importantly, the relevance of ACT may extend beyond helping parents tolerate stress in the moment. ACT processes such as values clarification, self-as-context, and committed action may help parents interpret PA support as an expression of the kind of parent they want to be, thereby strengthening an identity around encouraging, facilitating, and joining in their child’s PA (Grant et al., 2025; Hayes et al., 2006; Strachan et al., 2026). This identity-focused rationale is compatible with the Multi-Process Action Control (M-PAC) framework (Rhodes, 2021), which distinguishes reflective processes such as perceived capability and affective judgments, regulatory processes such as planning and self-monitoring, and longer-term reflexive processes such as identity and habit (Rebar & Rhodes, 2020; Rhodes, 2021; Rhodes et al., 2016a). In the present intervention logic, ACT was used to address the emotional and values-based conditions under which parental support is enacted, while M-PAC informed the action control structure needed to translate supportive intentions into repeated behaviour. More specifically, self-as-context and values clarification were intended to support PA-support identity by helping parents view supportive actions as expressions of valued parenting (Hayes et al., 2006; Strachan et al., 2026), defusion and self-compassion targeted self-critical thoughts and affective barriers that could undermine perceived capability and follow-through (Masuda et al., 2004; Neff, 2003), and committed action, planning, and self-monitoring connected values to M-PAC regulatory processes (Hayes et al., 2006; Rhodes, 2021). Applied to parental support for child PA, ACT may therefore complement M-PAC by helping parents use reflective motivation and regulatory strategies under emotional pressure, while repeated values-consistent enactment may support more stable PA-support identity and habit over time (Pears & Sutton, 2021; Strachan et al., 2026; Zhang et al., 2018). Thus, ACT and M-PAC were treated as complementary frameworks: ACT clarified how parents might remain psychologically flexible under emotional pressure, whereas M-PAC specified the reflective, regulatory, and reflexive processes through which supportive behaviour may be initiated, enacted, and maintained. However, to our knowledge, no intervention research has directly tested this combined ACT-informed and M-PAC-compatible rationale in the specific context of helping parents support their children’s PA, despite its clear theoretical relevance.

### The Present Study

Guided by the Obesity-Related Behavioral Intervention Trials (ORBIT) model (Czajkowski et al., 2015), this study was positioned as a Phase IIa proof-of-concept evaluation of a remotely delivered, ACT-informed emotion regulation program designed to help parents support their children’s PA. Consistent with the early-phase aims of ORBIT, the purpose of the present study was not to establish efficacy, but to determine whether this theory-driven intervention showed sufficient promise to warrant further refinement and later-phase testing. Since this was a qualitative Phase IIa proof-of-concept study, no formal a priori quantitative progression criteria were specified. Instead, progression to further optimization or later-phase evaluation was to be guided by whether the interview data indicated sufficient acceptability, perceived feasibility, promising perceived impacts on parental support for child PA, and clear directions for refining an optimizable intervention package. Accordingly, the study was guided by the following research question: How did parents perceive the acceptability, feasibility, perceived impact, and refinement needs of the remotely delivered ACT-informed emotion regulation program for supporting parental support of children’s PA?

## 2. Materials and Methods

### 2.1. Design

Following the ORBIT framework (Czajkowski et al., 2015), this study was designed as a single-arm, Phase IIa proof-of-concept study of a remotely delivered, ACT-informed emotion regulation intervention intended to support parents in promoting their children’s PA. Remote delivery was intentional at this early stage because parents’ experiences of family-based PA interventions are shaped by delivery demands, available resources, and contextual barriers to engagement, while broader parenting-intervention research suggests that web-based delivery and practical supports may enhance parent engagement when participation burden is manageable (Aldridge et al., 2024; Brennan et al., 2025). Reporting was informed by the CONSORT extension for pilot and feasibility trials (Eldridge et al., 2016), adapted for a non-randomised design with randomisation-specific items treated as not applicable (Lancaster & Thabane, 2019). Qualitative exit interviews were used to explore parents’ perceptions of acceptability, perceived feasibility, and promising signals of perceived impact, with the broader goal of informing intervention refinement and later-phase testing. Ethical approval was granted by the first author’s institutional Human Research Ethics Board (#25-0010).

### 2.2. Participants

Participants were parents or guardians residing in Canada who had at least one child aged 6 to 12 years who, according to parent report during screening, was engaging in less than 60 min per day of MVPA. This age range was selected because children in this age range remain highly dependent on parents for practical and motivational support for PA, making this period particularly relevant for an intervention designed to strengthen parental support behaviours (Rhodes et al., 2020a, 2024). Parents of any gender, ethnicity, or socioeconomic background were eligible if they could communicate in English and had access to the internet and a device to participate in the online workshops. A target sample of 15 parents was set a priori for this Phase IIa proof-of-concept study. This target was judged appropriate for a focused qualitative study because the research aim was narrow, the participant group was specific, and the interviews were theory-informed and expected to yield information-rich accounts (Hennink & Kaiser, 2022). Consistent with information-power principles, such conditions can support modest qualitative samples (Malterud et al., 2016), and empirical work suggests that focused interview studies with relatively homogeneous samples commonly identify recurring thematic issues within approximately 9 to 17 interviews (Hennink & Kaiser, 2022). Since the sample size was set a priori and exit interviews were conducted with workshop completers, saturation was not used to determine recruitment stopping. During analysis, however, later interviews produced substantially similar feedback around the workshops’ acceptability, the feasibility of applying strategies in daily family life, the usefulness of flexible rather than all-or-nothing support, and the need for refinements such as sustained engagement supports and greater interactivity. We therefore judged sample adequacy by both the richness of participants’ accounts and the recurrence of cross-case themes sufficient to support coherent theme development. Recruitment was conducted Canada-wide using advertisements distributed primarily through social media platforms, including Instagram and Facebook. Interested parents contacted the research team by email and were screened for eligibility based on their child’s age and daily MVPA. Eligible parents were then provided with the study consent form by email, and those who consented received the Zoom links for the workshop series.

### 2.3. Procedures

Families who expressed interest in the study contacted the research team by email. Initial screening was then completed by email and focused on the child’s age and parent-reported daily MVPA to determine eligibility. Eligible parents were provided with the consent form by email, and those who consented received Zoom links for the intervention. The intervention consisted of three parent-only workshops, each lasting 20 to 30 min, delivered remotely over a three-week period. The workshops were delivered by the first author, whose background spans kinesiology, health psychology, behavioural medicine, and health promotion, and who has experience designing and delivering PA behaviour-change interventions. Then, 1 to 2 weeks after the third workshop, participants completed a one-on-one exit interview on Zoom with the second author, who had not delivered the intervention workshops, to explore the program’s acceptability, feasibility, and perceived impact. Families attended the online sessions individually, and no incentives, reimbursement, or compensation were provided for participation.

### 2.4. Intervention Materials

To support delivery consistency and fidelity, all participants received the same intervention package, consisting of three individual Zoom workshops delivered using structured slide decks and accompanied by take-home worksheets. Across the three sessions, the intervention combined behavioural medicine-oriented education on child PA and parental support with ACT-informed emotional regulation content and M-PAC-informed action control strategies. The sequence was deliberately progressive. Detailed intervention materials and corresponding Behaviour Change Technique Ontology (BCTO) terms with BCIO identifiers are presented in Table 1 (Marques et al., 2024).

The first workshop served as the educational, motivational, and planning foundation of the intervention (Lee et al., 2025; Medd et al., 2020; Streight et al., 2024). After orienting parents to the study timeline, workshop sequence, and take-home worksheets, the session introduced the behavioural target through education on the Canadian 24-Hour Movement Guidelines for Children and Youth, including the recommendation of at least 60 min per day of MVPA (Tremblay et al., 2016), and the physical-health, developmental, emotional, and family benefits of PA. In BCTO terms, this content primarily involved informing about positive health and emotional consequences. Parental support was then defined as encouragement, logistical support, and co-participation (Pyper et al., 2016; Rhodes et al., 2020b; Yao & Rhodes, 2015), with concrete examples such as emphasizing fun, creating activity opportunities, validating movement as a family priority, and joining the child in walking or play; this reflected instructing parents how to perform support behaviours. The session then shifted to why parents may struggle to follow through despite good intentions, highlighting stress, fatigue, and feeling overwhelmed as emotional barriers to support behaviour (Bassett-Gunter et al., 2017; Maher et al., 2017). This discussion set up the introduction of ACT as a values-based approach for helping individuals remain engaged in meaningful action even in the presence of difficult thoughts and feelings (Flujas-Contreras et al., 2024; Hayes et al., 2006). Parents completed a values clarification and identity-reflection exercise focused on the kind of parent they wanted to be around their child’s PA, how that identity could be expressed in daily life, and what lower-priority demands might need to be displaced (Lee et al., 2025; Streight et al., 2024). In BCTO terms, this segment most closely reflected affirming valued self-identity, identifying oneself as a role model, and, where parents reconsidered support as an expression of valued parenting, reframing past or current support behaviour. The workshop concluded with ACT-consistent committed action through a commitment statement, behaviour-goal setting, action planning, and goal strategizing, as parents developed concrete what/where/when/how plans for supporting their child’s PA (Hayes et al., 2006; Rhodes et al., 2019a, 2016b). Take-Home Worksheet 1 extended this content into daily life by asking parents to reflect on emotions that shaped support behaviour, clarify values and a value mantra, write a commitment statement, and develop a specific support plan (Grant et al., 2025; Lithopoulos et al., 2020; Rhodes et al., 2019a).

The second workshop shifted from rationale and planning to emotional regulation practice, with the goal of helping parents respond more flexibly when difficult internal experiences threatened to interrupt support for their child’s PA. The session began by reviewing Worksheet 1, which encouraged parents to notice how emotions shaped their support behaviour across the previous week; in BCTO terms, this reflected self-monitoring of behaviour. The workshop then taught four ACT-based skills through brief explanation, guided practice, and take-home application: acceptance through the NAME framework (**N**otice, **A**cknowledge, **M**ake room, **E**xpand awareness) (Harris, 2019), present-moment awareness through a short breathing exercise and the ACE framework (**A**cknowledge thoughts and feelings, **C**ome back into your body, **E**ngage with the world) (Harris, 2019), self-compassion through a guided reframe of a perceived support “failure,” and defusion through the phrases “I’m having the thought that…” and “Thank you, mind…”(Flujas-Contreras et al., 2024; Grant et al., 2025; Hayes et al., 2006; Kabat-Zinn, 1994; Masuda et al., 2004; Neff, 2003, 2023). In BCTO terms, these activities primarily involved practising behaviour and advising cognitive or behavioural ways to reduce negative emotions, while remaining ACT-consistent by emphasizing openness, awareness, and values-guided action rather than suppression or avoidance (Masuda et al., 2004; Neff, 2003; Sirois et al., 2019). The closing emotional-barriers exercise used goal strategizing: parents identified a recent emotionally difficult support situation, recorded the trigger, emotion, and behavioural response, and considered how acceptance, mindfulness, self-compassion, or defusion could support a different response in the future. Take-Home Worksheet 2 extended these skills into daily parenting situations by prompting practice with present-moment awareness, acceptance, and defusion between sessions (Grant et al., 2025; Hayes et al., 2006; Kabat-Zinn, 1994; Masuda et al., 2004).

The third workshop emphasized enactment and maintenance by helping parents convert their values and emotional regulation skills into sustained support behaviour under imperfect real-world conditions. It opened by revisiting Worksheet 2, after which parents completed a 0-to-10 self-rating of psychological flexibility across the ACT domains of opening up, being present, and doing what matters. In BCTO terms, this functioned as self-monitoring of behaviour by helping parents identify where future support actions were most vulnerable to breakdown (Hayes et al., 2006). The workshop then introduced practical strategies to strengthen follow-through. Positive self-talk was taught through parent-generated phrases for moments of fatigue, stress, or time pressure, reflecting prompt self-talk (Latinjak et al., 2023). Visualization was framed as mentally rehearsing a difficult support situation and successfully navigating it, reflecting prompt mental rehearsal of successful performance (Grant et al., 2025; Liu et al., 2025). Implementation intentions were taught as if-then planning, reflecting action planning, with parents linking recurring barriers to specific responses such as taking a few breaths, using a coping phrase, or completing a shorter bout of co-activity (Divine & Astill, 2025; Hagger et al., 2016). Avoiding all-or-nothing thinking extended this work through goal strategizing, as parents generated lower-intensity or shorter-duration alternatives rather than abandoning support when the original plan became unrealistic (Segar et al., 2026). Finally, mental toughness was introduced as a maintenance-oriented concept to reinforce persistence and recovery after setbacks, with prompts around flexibility, strength, and resilience (Dziuba et al., 2025; Gucciardi, 2017). This workshop positioned consistency over perfection and emphasized that values-guided support could still occur in modified form when motivation, energy, or circumstances were suboptimal.

### 2.5. Qualitative Data Collection

After completing all three workshops, participating parents completed an individual semi-structured exit interview conducted by the second author via Zoom. Interviews were audio-recorded for transcription and were guided by a structured interview schedule adapted from Bowen et al. (2009) to assess feasibility-related domains. The guide used workshop-specific prompts to cue parents’ recall of the intervention content and then asked common questions about the workshops’ acceptability (e.g., “How did you feel about the workshops? Please mention anything relating to the design of the slides, the information presented, the aesthetics of the presentation, or anything else you find relevant”), perceived demand and relevance (e.g., “Do you feel there is demand for this study? Please comment on how relevant and in demand you felt about the material”), feasibility in daily life (e.g., “Is this program feasible to implement in your daily life?”), ease of implementation (e.g., “Can you easily carry the workshop recommendations in your daily life?”), adaptability to unexpected events (e.g., “Can you modify these lessons if unexpected events in your life come up quickly?”), integration of strategies (e.g., “How easily can you integrate these lessons (for example, mindfulness and defusion) into your daily lives?”), fit with family dynamics (e.g., “Do the ideas from this study, overall, fit within the dynamics of your family?”), and the perceived novelty or insightfulness of the material (e.g., “Did you find the material presented in the workshops new and innovative? Were there any concepts or strategies that you had not heard of before or found particularly insightful?”). In keeping with the qualitative proof-of-concept focus of the present study, these exit interviews constituted the principal source of outcome-relevant data. The full exit interviews schedule is presented in Appendix A.

### 2.6. Data Analyses

All exit interviews were audio-recorded and transcribed for analysis. The qualitative data were analyzed using cross-case thematic analysis, with Microsoft Excel used to organize transcripts, initial codes, analytic notes, candidate categories, and developing themes (Braun & Clarke, 2006; Kiger & Varpio, 2020). The interviews were treated as a single analytic dataset, and the analysis focused on identifying patterned meanings across participants’ accounts instead of producing stand-alone summaries of individual cases.

The analysis was primarily semantic and data-near, with coding focused first on participants’ explicit descriptions of how they experienced the workshops, used the strategies, and interpreted the intervention in daily life, rather than beginning from a strongly theory-driven or highly latent reading of the data (Braun & Clarke, 2006; Sandelowski, 2010). Accordingly, the first author read and re-read all transcripts and generated codes primarily inductively from participants’ accounts, while sensitized by the feasibility domains that structured the interview guide and the ACT/M-PAC constructs relevant to the study aims; these frameworks guided analytic attention but did not operate as a fixed deductive coding template (Bowen et al., 2009; Braun & Clarke, 2006; Kiger & Varpio, 2020). Related codes were then compared across transcripts, grouped into candidate categories, and organized into subthemes and overarching themes that captured recurring patterns in acceptability, feasible application, perceived impact, and refinement needs (Braun & Clarke, 2006; Kiger & Varpio, 2020). Particular attention was paid not only to acceptability and fit, but also to perceived mechanisms of change, translation of the material into real-world family life, negative or deviant cases, and implications for intervention refinement. Direct quotations were retained and labeled by interview number to support transparency in the analytic write-up. Reflexively, the first and third authors’ involvement in designing the ACT- and M-PAC-informed intervention logic provided close familiarity with the intended rationale, and the first author’s additional role in delivering the workshops and leading the analysis created the possibility of overemphasizing interpretations that aligned with that rationale. To reduce this risk, coding and theme development prioritized participants’ explicit accounts, including critical, ambivalent, and deviant cases. Developing interpretations and theme boundaries were then discussed with the second author, who conducted the interviews but did not deliver the workshops, and the third author to challenge assumptions, refine the organization of themes, and strengthen the credibility of the final analysis, consistent with qualitative recommendations to examine disconfirming cases and use analytic discussion to strengthen interpretation (Patton, 2002).

## 3. Results

### 3.1. Sample Characteristics

Of 57 potential participants screened or assessed for eligibility, 27 were ineligible and 30 were eligible. Of the 30 eligible parents, 9 did not consent and 21 began the intervention by attending Workshop 1. Seventeen parents attended Workshop 2, and 15 completed the workshop series by attending Workshop 3, yielding a workshop completion rate of 71.4% among those who began the intervention. Twelve of the 15 workshop completers completed the exit interview, yielding an exit-interview completion rate of 80.0% among workshop completers and an overall completion rate of 57.1% from intervention start to exit interview. The qualitative sample comprised 11 mothers and 1 father and included parents from five Canadian provinces: Ontario (*n* = 5), Alberta (*n* = 3), British Columbia (*n* = 2), Quebec (*n* = 1), and Manitoba (*n* = 1). The exit interview completers represented families with one to three eligible children aged 6 to 12 years, with 17 children in this target age range represented across families (M = 8.85, SD = 1.93).

### 3.2. Qualitative Analysis

**Figure 1:**
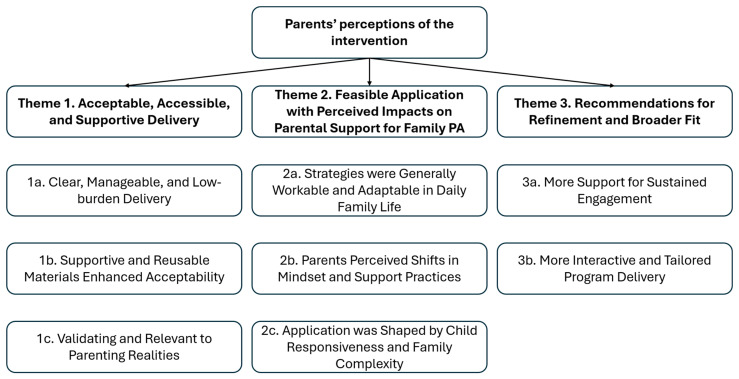
Thematic map of parents’ perception of the ACT-informed emotion regulation program. Note. The figure summarizes the three overarching themes and eight subthemes generated from the cross-case thematic analysis.

Cross-case thematic analysis generated three overarching themes and eight subthemes that captured parents’ views of the intervention’s acceptability, feasible application and perceived impacts, and directions for refinement and broader fit (Figure 1; Table 2).

### 3.3. Theme 1. Acceptable, Accessible, and Supportive Delivery

This theme comprised three subthemes and captured how parents experienced the workshop series as a delivered intervention package. At a broad level, it reflected parents’ views of the program as clear and manageable, supported by useful materials, and delivered in a way that felt responsive to the emotional realities of parenting.

**Clear, Manageable, and Low-burden Delivery**. Most parents described the workshop series as acceptable because it was clear, manageable, and not overly burdensome. Acceptability was therefore reflected not only in general satisfaction, but also in the sense that psychologically oriented material was presented in an approachable format. For example, one parent described the workshops as “really efficient” and said participation “didn’t take a lot of my time or energy” (Interview 08). Another parent similarly felt the workshops were “very, very well done,” “not too long,” and “a good length,” adding, “I enjoyed it. I learned a lot” (Interview 04). Minor critiques were framed as refinements rather than dissatisfaction. Interview 04, for instance, suggested that some slides were “a bit on the busy side” and could be split across multiple slides to reduce distraction. Overall, parents appeared to view the workshops as appropriately pitched in length, complexity, and delivery burden, while identifying limited opportunities to improve visual clarity.

**Supportive and Reusable Materials Enhanced Acceptability**. Acceptability was also strengthened by the fact that the materials extended beyond the live sessions. Several parents described the slides and worksheets as resources they could revisit afterward, which reduced pressure to retain everything in real time and helped connect the workshop content to daily life. Interview 11 captured this connection, explaining that having the slides afterward was “helpful” because the material was “easy to understand and easy to apply to a family situation,” and that the worksheets were useful because they “directly correlated” with the workshop content rather than feeling disconnected from it. Similarly, Interview 12 said that receiving the workshop slides afterward meant they “didn’t have to worry about trying to retain that information,” allowing them to focus more fully on the presentation. Together, these accounts suggest that reusable and coherent materials enhanced acceptability by making the intervention feel more supportive, practical, and easier to absorb.

**Validating and Relevant to Parenting Realities**. A further feature of acceptability was that parents experienced the workshops as supportive and validating rather than merely informational. Parents described feeling recognized in the emotional realities of trying to support PA within family life. Interview 11 described the sessions as “that combination of support and education,” explaining that they felt “validated” by both the workshop discussion and the slides, and later added that the program helped them feel they “didn’t feel alone in the process of trying to implement this with my children.” Interview 12 similarly described the self-compassion content as “something that I needed to hear,” particularly as a parent who tends to be constantly “go, go, go” and can “forget about the me piece.” Interview 05 also valued that the sessions involved conversation, questions, and suggestions rather than passive presentation alone. Together, these accounts suggest that the workshops were acceptable not only because they were clear and well organized, but because they were delivered in a way that helped parents feel understood without being judged.

### 3.4. Theme 2. Feasible Application with Perceived Impacts on Parental Support for Family PA

This theme comprised three subthemes and focused on what happened beyond the workshops themselves. It captured parents’ accounts of how the strategies were used in daily family life, the shifts they perceived in their thinking and support practices, and the conditions under which implementation became more difficult.

**Strategies were Generally Workable and Adaptable in Daily Family Life**. Most parents described the strategies as workable within everyday family life, particularly when they could be applied flexibly rather than perfectly. The most common example was avoiding all-or-nothing thinking by scaling activity down instead of abandoning it. Interview 08 explained this as changing plans in real time: “Oh, it actually is quite hot out. So we’ll shorten our ride, or we’ll ride to the beach instead… Even if we get out for a 10 min walk after dinner, that’s better than nothing, even if I had planned to go for a run with them.” Interview 04 described a similar pattern, reducing expectations on difficult days from 30 min to 10 min and substituting alternative activities when original plans were no longer realistic. Parents also described using self-regulation strategies to support follow-through, including deep breathing, mindfulness, and present-moment awareness; Interview 10 identified “avoiding [the] all-or-nothing thinking” and “just being in the moment” as the strategies they would “more use first.” Feasibility also depended on adapting family routines. For example, Interview 01 explained that if both parents could not participate, they would “divide our work in between” so that “one parent can go” and the child would not have to “skip the play-date outside.” These accounts suggest that feasibility was expressed through identifiable strategies, including scaling plans down, substituting activities, using self-regulation tools, and reorganizing routines so support for PA could still occur under less-than-ideal conditions.

**Parents Perceived Shifts in Mindset and Support Practices**. Parents also described perceived shifts in how they thought about supporting family PA, particularly around flexibility, emotional awareness, and expectations. Interview 08 offered one of the clearest examples, explaining that “the biggest one for me” was “recognizing flexibility and letting go of the all-or-nothing thinking that I applied to myself,” and then applying that flexibility to helping their children be active. Interview 09 similarly described realizing that the self-talk they had previously used for their own exercise also applied to supporting their son: “the self-talk is the exact same. And I didn’t actually realize how much overlap there was until I started intentionally doing it.” For some parents, the perceived shift involved reframing the meaning of PA support itself. Interview 11 described moving away from being “almost hyper-fixated on perfection” toward a broader focus on “promoting physical and mental health within our children,” while Interview 10 said the study highlighted “the effect that their emotions” could have on their children’s active lifestyle. Across these accounts, parents did not describe dramatic behavioural transformation; rather, they described a modest but meaningful shift toward less pressure-based, less perfectionistic, and more emotionally aware support practices.

**Application was Shaped by Child Responsiveness and Family Complexity**. At the same time, parents made clear that feasibility for the parent did not automatically mean easy implementation at the family level. In several interviews, the main barrier was not parents’ willingness to use the strategies, but whether the child would engage and whether the approach fit the family context. Interview 06 captured this directly: “I think it is [feasible]. But again, I think me implementing it and getting results is sometimes a little bit difficult. You still need the other, the child to agree to it.” A few parents therefore suggested that future versions might include a workshop or component “more catered towards the child,” rather than leaving the parent to carry the implementation alone. Interview 07 highlighted family complexity as another boundary condition, explaining that the study did not fully capture households with “multiple different dynamics, like neurodiversity or disabilities,” including three neurodivergent children, chronic pain, and conflicting child preferences. Importantly, this was not experienced as a complete mismatch: the parent described the “base teachings” as helpful, while noting that “the problem solving aspect of it isn’t quite there yet.” These interviews suggest that feasibility was conditional rather than universal; parents often found the strategies meaningful, but their application depended on child readiness, developmental fit, disability- and neurodivergence-related needs, and the broader support structure within the family.

### 3.5. Theme 3. Recommendations for Refinement and Broader Fit

This final theme comprised two subthemes and brought together parents’ suggestions for strengthening the intervention in future iterations. These recommendations pointed to ways of improving ongoing engagement and making the program more interactive, flexible, and responsive to a wider range of family contexts.

**More Support for Sustained Engagement**. Although parents generally viewed the intervention positively, many treated the proof-of-concept format as a strong starting point rather than a finished program. One clear area for refinement was greater support for sustained engagement over time. Several parents suggested that the workshops would be strengthened by opportunities for reinforcement, review, and follow-through after the initial sessions. Interview 04, for example, recommended turning the slides into short videos that parents could revisit, explaining that they could then “go back and… review them whenever they want.” Interview 05 emphasized the need for active prompting, suggesting that reminders, possibly through app-based notifications rather than email, could help users “stay engaged in this commitment that they have made.” The same parent also suggested slower pacing, such as spacing sessions every 2 weeks and adding reminders every 3 to 4 days between sessions. Other parents pointed to similar maintenance needs through comments about practice and accountability; Interview 09 suggested that “other families, or more social aspect” might help with the “different accountability part” of sustaining new habits. According to these interviews, parents were not only evaluating short-term usefulness, but also identifying ways the program could better support retention, repetition, and behavioural maintenance once the initial workshops had ended.

**More Interactive and Tailored Program Delivery**. A second set of recommendations concerned making the program more interactive, practical, and responsive to differences across families. Some parents felt that, because the target behaviour was PA, delivery would benefit from more demonstration or practice. Interview 05 noted that a fully online format could create “a gap or a distance” and suggested that an in-person or additional practical component might help parents see strategies “in action.” Other parents emphasized peer exchange. Interview 09 recommended online group sessions with “six to ten parents” to allow a “deeper dive” into specific examples and shared problem-solving. Broader fit also appeared to require more child-facing and tailored content. Interview 06 suggested that future versions might include a workshop “more catered towards the child,” so that implementation would not rest solely on the parent. Parents also raised linguistic and cultural tailoring, including translation into other languages and adaptation for different diaspora communities. These recommendations suggest that the next-stage intervention should be more participatory, flexible in format, inclusive of children where appropriate, and responsive to the diverse family contexts in which PA support is enacted.

## 4. Discussion

This proof-of-concept study examined the acceptability and perceived feasibility of an ACT-informed emotion regulation program for parents, and explored parents’ perceptions of whether the program helped them manage emotional barriers to supporting their child’s PA. Overall, the findings suggest preliminary promise: parents generally viewed the program as acceptable, described the strategies as workable and meaningful in daily family life, and identified clear directions for refinement and broader fit. This pattern is important because it aligns with broader evidence that the central challenge in parental support for child PA is often not lack of knowledge or intention, but sustaining support under the emotional and practical pressures of everyday family life (Bassett-Gunter et al., 2017; Lithopoulos et al., 2020; Rhodes et al., 2016b). In that respect, the findings provide early qualitative support for the intervention’s ACT-informed emotion regulation rationale, while also being broadly consistent with the M-PAC-informed reflective, regulatory, and reflexive elements built into the program (Hayes et al., 2006; Rhodes, 2021).

To our knowledge, this is among the first qualitative proof-of-concept studies to examine an ACT-informed emotion regulation intervention specifically designed to help parents support their children’s PA, which helps explain why an early-phase ORBIT design was appropriate. At the same time, consistent with ORBIT Phase IIa aims, these findings should be interpreted as evidence of promise and intervention optimization needs rather than efficacy (Czajkowski et al., 2015; Eldridge et al., 2016). The following sections interpret the findings in terms of theoretical alignment, applied fit with parenting realities, and optimization needs prior to later-phase evaluation.

### 4.1. Theoretical Alignment of ACT and M-PAC

The most theoretically informative finding was not simply that parents viewed the strategies as feasible, but that some accounts suggested a more flexible way of judging support as worth enacting. The interviews do not mainly suggest that parents left the program with stronger intentions to support their child’s PA; rather, some parents described treating shortened, substituted, or lower-intensity activities as worthwhile forms of support when the original plan was no longer realistic (Grant et al., 2025). This interpretation fits the broader parental support literature, where the recurring challenge is not only endorsing support in principle, but enacting and sustaining it when family life becomes effortful, inconvenient, or emotionally taxing (Brown et al., 2016; Rhodes & Lim, 2018). Maintenance theories therefore place considerable weight on adaptation, coping, and recovery processes, not only on motivation or intention strength (Kwasnicka et al., 2016; Zhang et al., 2018). Viewed through that lens, the prominence of avoiding all-or-nothing thinking in the present data is theoretically informative. It suggests that the intervention may have helped some parents redefine successful support less in terms of executing the original plan perfectly and more in terms of maintaining values-consistent action in an adapted form when conditions were unfavorable, which aligns closely with ACT’s emphasis on committed action and with implementation-focused approaches to follow-through (Hagger et al., 2016; Hayes et al., 2006; Rhodes, 2021).

This is where the ACT-informed and M-PAC-informed elements of the intervention appear conceptually symmetrical: ACT offers a way to remain open to difficult internal experiences while still acting in line with values, whereas M-PAC specifies how regulatory processes can protect intention enactment and support repeated behaviour under challenging conditions (Hayes et al., 2006; Rhodes, 2021). The interviews therefore point to something more specific than general motivational uplift: several parents described becoming less perfectionistic, less harsh with themselves, and more able to keep supporting their child’s PA in workable, scaled forms despite stress, fatigue, frustration, or self-critical thoughts, which is much more consistent with psychological flexibility and self-compassion than with motivation alone (Neff, 2003, 2023; Pears & Sutton, 2021).

Beyond flexible enactment, some parents appeared to reinterpret PA support as part of who they were trying to be as parents, rather than merely as a task to complete more efficiently. Several accounts moved beyond tactical adjustment and pointed to a broader shift in self-understanding: exercise-related self-talk that had previously been used in relation to one’s own activity was newly recognized as relevant to parenting; perfectionistic standards were loosened; support was no longer framed only as “getting the family moving,” but as contributing to the child’s physical and mental well-being and to the kind of family climate the parent wanted to cultivate (Brennan et al., 2025). This reframing is theoretically relevant. In ACT terms, it is consistent with values clarification, self-as-context, and committed action, all of which are intended to reorganize behaviour around chosen directions rather than transient frustration, guilt, or self-judgment (Hayes et al., 2006). It also gives M-PAC a more precise supporting role than simple action control (Rhodes, 2021).

Previous identity-related literature (Rhodes, 2021; Strachan et al., 2026) argues that repeated behaviour becomes more stable when it is integrated into identity-relevant self-views; the present findings suggest that this process may begin not only when parents repeat support behaviours, but when they start to understand those behaviours as expressions of valued parenting. Grant et al. (2025) showed that affect regulation, identity, and valued living can shift in adults managing their own PA. The present study suggests that similar processes may be recruited in a more relational domain, where the behavioural target is not one’s own exercise but the ongoing support of a child’s activity within family life. These findings suggest that the intervention’s early promise may lie not only in helping parents persist when conditions are difficult, but also in helping them integrate PA support into parental identity in a way that may make future enactment more coherent, less self-punitive, and potentially more durable over time (Hayes et al., 2006; Lithopoulos et al., 2020; Rhodes, 2021; Strachan et al., 2026).

### 4.2. Applied Fit: Acceptability, Delivery, and Parenting Realities

Whereas the previous section highlights the theoretical significance of the perceived shifts in enactment and identity, the applied significance of the findings lies in how this psychologically focused material was delivered. Acceptability in this study was not simply a matter of parents finding the workshops pleasant or convenient; instead, it suggests that emotionally focused, ACT-informed material could be translated into a form that parents experienced as sufficiently concrete, low-burden, and family-relevant to engage with.

Previous parent PA-support interventions have often centered education, planning, and logistical problem solving (Brown et al., 2016), whereas the present program asked parents to engage with more psychologically demanding content such as acceptance, self-compassion, defusion, and values-based reflection, all of which could easily have been experienced as too abstract, too clinical, or too burdensome if poorly packaged (Bassett-Gunter et al., 2017; Hayes et al., 2006). The present findings suggest that brief remote delivery, coherent worksheets, and practical examples helped convert those ideas into something parents saw as usable in family life, which is highly relevant in an early-phase ORBIT context where intervention promise depends not only on theoretical logic but on whether participants can realistically receive and tolerate the intervention as designed (Bowen et al., 2009; Brennan et al., 2025; Czajkowski et al., 2015; Eldridge et al., 2016). In that sense, the current findings sit well alongside adjacent evidence that affect- and ACT-informed content can be delivered digitally in PA and parent-focused contexts, including Grant et al. (2025)’s web-based affect-regulation PA trial and Flujas-Contreras et al. (2024)’s internet-based ACT work with parents, while extending that literature into the more specific and underexamined context of parental support for children’s PA.

This applied fit was not only a matter of brevity or convenience. A crucial feature of delivery was that parents experienced the workshops as supportive and validating rather than prescriptive. That distinction matters because the target problem in this study was not parental ignorance about PA guidelines, but the emotional friction surrounding follow-through, including guilt, frustration, fatigue, and pressure, and a delivery style that intensified judgment would likely have worked against the intervention’s own ACT-consistent aims (Bassett-Gunter et al., 2017; Hayes et al., 2006; Neff, 2003). From that perspective, the validating tone was not merely a presentation strength; it may have been part of the intervention’s functional mechanism, because parents may be more willing to engage in acceptance, self-compassion, and values-guided action when the intervention itself models those same qualities (Hayes et al., 2006; Neff, 2003). A related caution is that the acceptability observed here may have been tied closely to the specific delivery conditions of the program. The workshops were brief, individualized, and offered opportunities for clarification, yet some parents still described the slides as visually busy, suggesting that there were limits to how much material could be comfortably absorbed even in a relatively supportive format. These findings therefore should not be read as evidence that remote delivery is inherently acceptable. A narrower conclusion is warranted: this kind of parent-focused, ACT-informed content appears more acceptable when emotionally complex material is translated into concrete examples, supported with reusable materials, and delivered in a way that keeps cognitive load manageable while preserving warmth and interpretive guidance (Bowen et al., 2009; Grant et al., 2025).

Family fit also emerged as a boundary condition. The more complex accounts suggested that ACT-informed psychological flexibility may help parents respond less reactively to stress, guilt, or frustration, but it does not remove the need for child-specific problem solving when barriers are driven by child readiness, neurodevelopmental needs, disability, chronic pain, or conflicting family preferences. This interpretation is consistent with parent PA-support literature showing that support is shaped by child interest, available resources, and family context, not only by parental motivation or intention (Bassett-Gunter et al., 2017; Brennan et al., 2025; Rhodes & Lim, 2018). Thus, the optimization task is not to replace the parent emotion regulation core, but to strengthen the bridge between parent learning and family-level enactment.

### 4.3. Optimization Prior to Later-Phase Evaluation

The refinement findings suggest that the next developmental step is not to expand the intervention’s therapeutic content, but to strengthen the implementation structure around content that parents already viewed as meaningful. The refinement requests converged on two linked needs: first, parents wanted more support to retain and reuse the material after the initial workshops; second, they wanted a clearer bridge between parent learning and what can actually be enacted within family life.

Based on these findings, four refinements appear warranted before later-phase evaluation. First, the workshop slides should be converted into brief asynchronous review materials, such as short videos or visual summaries, so parents can revisit key ACT and planning strategies after the live sessions; this is consistent with technology-assisted parenting research in which web-based modules, videos, videoconferencing, emails, phone calls, and text or app messaging are commonly used delivery modes (Aldridge et al., 2024). Second, the program should include structured prompts between sessions, such as brief text, email, or app-based reminders every few days, to cue practice and maintain salience once initial motivation decreases; this aligns with behavioural intervention technology models that emphasize specifying not only intervention content but also the workflow and timing through which components are delivered (Mohr et al., 2014). Third, the pacing should be reconsidered, either by spacing the three workshops over a longer period or adding a brief booster contact after the final session, so parents have more time to practise, encounter barriers, and receive support for adaptation; this recommendation is consistent with evidence that human support may improve engagement and outcomes in digital interventions, while also requiring clear reporting of the function and mechanism of that support (Werntz et al., 2023). Fourth, the remote parent-focused core should be supplemented with more interactive and family-facing elements, such as brief child-facing content, parent–child practice examples, or small online group problem-solving sessions where parents can discuss specific barriers and adaptations (Brennan et al., 2025).

This proposed refinement is supported by qualitative synthesis work showing that parents’ experiences of family-based PA interventions are shaped by intervention components, delivery, social connections, child interest, and available resources, and that future interventions should provide opportunities for social support and strengthen parents’ PA-support identities (Brennan et al., 2025). These refinements should be treated as delivery and enactment supports rather than changes to the intervention’s active ingredients. Short videos, reminders, slower pacing, group discussion, and selective child-facing content would preserve the ACT-informed core if they continue to rehearse acceptance, defusion, present-moment awareness, self-compassion, values clarification, and committed action, while using M-PAC-informed planning, self-monitoring, and follow-through strategies to support repeated enactment (Hayes et al., 2006; Marques et al., 2024; Rhodes, 2021). Framed this way, the refinements preserve the scalability of a primarily remote model while aligning with family-system approaches that emphasize active family identity and involvement of family members in PA together (Lee et al., 2025; Streight et al., 2024).

### 4.4. Limitations and Future Directions

Several limitations should be considered when interpreting the findings. First, this was a single-informant qualitative evaluation in which the main outcome-relevant data came from parents’ own post-intervention accounts. Accordingly, the findings speak to parents’ perceived acceptability, feasibility, and impact rather than independently verified changes in parental support or child PA. Nor can the present design determine whether perceived shifts were driven by ACT-specific processes, M-PAC action control strategies, facilitator support, structured reflection, planning exercises, or some combination of these elements. A subsequent fully powered trial would be needed to test these mechanism questions directly, using additional informants, especially children and, where relevant, other caregivers, together with quantitative indicators of parental support, candidate mechanisms of change, and child or family PA. Concurrent exposure to other parenting, PA, or behaviour-change programs was not measured in the present study. Future randomized trials should address these exposures prospectively, either through eligibility criteria or as baseline/contextual descriptors, to support interpretation of feasibility and outcome signals.

Second, although most workshop completers also completed the exit interview, attrition occurred across the full intervention pathway, meaning that the qualitative findings may overrepresent parents who were more motivated, more satisfied with the program, or more able to engage with the workshops and exit interview. This is important for future trial design because parent-facing PA programs must compete with substantial family, time, and delivery demands, and parents’ engagement is shaped by available resources, child needs, and contextual barriers (Brennan et al., 2025). Future studies should incorporate retention supports such as flexible scheduling, brief reminders, asynchronous review materials, and systematic tracking of reasons for noncompletion, so feasibility can be evaluated across the full intervention pathway rather than only among completers.

Third, sample identification and transferability should be considered. The qualitative sample was self-selected and recruited primarily through social media, which may have attracted parents who were already interested in child PA, parenting support, or emotion regulation strategies. Eligibility also relied on parent-reported child MVPA during screening, which reduced recruitment burden for this remote proof-of-concept study but may have introduced recall or social desirability error and misclassified some children’s guideline adherence. Future trials should use a brief validated child PA measure, such as the PAQ-C (Kowalski et al., 1997), and/or accelerometry where feasible (Streight et al., 2024), to screen eligibility, characterize baseline activity, and improve interpretation of intervention-related change. Although the sample included parents from five Canadian provinces, it did not include families from Atlantic Canada or the Canadian territories, limiting confidence that the findings capture region-specific barriers or delivery needs across the full Canadian context. Future studies should seek broader regional representation, particularly if the intervention is intended for Canada-wide remote delivery, and should examine whether the program remains acceptable and feasible among parents with supportive intentions but different levels of readiness, time availability, delivery preferences, and familiarity with psychologically oriented parenting content.

Fourth, the sample was heavily weighted toward mothers, with only one father represented. This limits confidence in how well the findings reflect fathers’ experiences, particularly given evidence that fathers can play an important role in children’s PA through encouragement, facilitation, role modelling, and co-participation (Zahra et al., 2015). At the same time, mother-heavy samples are common in parent–child research and may reflect an important route of parental reach, because mothers are often central providers of PA support and may view this support as part of their parental role (Brennan et al., 2024; Rhodes et al., 2019b). Future studies should therefore retain strategies that successfully reach mothers while also making more deliberate efforts to recruit and retain fathers and other caregivers.

Fifth, although delivery consistency and fidelity were supported through standardized slide decks, worksheets, and mapped intervention components, the study did not include an independent fidelity assessment. Future studies should incorporate a fidelity protocol aligned with the intervention logic and BCTO-mapped components.

Finally, the evaluation was short-term. Parents were interviewed 1 to 2 weeks after the final workshop, so the study cannot determine whether the reported shifts were maintained over time. Future later-phase work should retain the current emotion regulation core while refining delivery features identified in this study, including supports for sustained engagement and clearer pathways from parent learning to family-level enactment. Before proceeding to a definitive randomized controlled trial, future studies should pre-specify feasibility progression criteria across recruitment, retention, workshop completion, delivery fidelity, acceptability, and measurement completion. The outcome battery should also be selected or adapted for this specific context, particularly for parental PA support and psychological flexibility in situations involving stress, fatigue, child resistance, and competing family demands (Brennan et al., 2025). Candidate mechanism measures, such as psychological flexibility, self-compassion, emotion regulation, parental perceived capability or self-efficacy, and PA-support identity, may then be incorporated into a subsequent fully powered trial to test whether changes in these processes explain changes in parental support and child or family PA (Hayes et al., 2006; Neff, 2003; Rhodes, 2021; Zhang et al., 2018).

## 5. Conclusions

This proof-of-concept study suggests that a brief, remotely delivered ACT-informed emotion regulation program was acceptable to participating parents and showed early qualitative promise for helping them manage emotional barriers to supporting their child’s PA. Parents described the program as understandable, feasible to apply in daily family life, and potentially helpful in fostering more flexible, less perfectionistic, and more values-consistent support practices. At the same time, the findings point clearly to the need for intervention optimization, particularly through stronger support for sustained engagement and more direct ways to translate parent learning into family-level uptake, including child inclusion where appropriate. Next-stage work should refine the delivery features identified here, pre-specify feasibility progression criteria, and select or adapt context-appropriate measures of parental PA support, psychological flexibility, and child or family PA for later-phase evaluation.

## Figures and Tables

**Table 1:** Intervention Materials.

Workshop	Title	Content	BCTO Terms and ACT Constructs	Corresponding M-PAC Process
1	Understanding Emotions to Support an Active Family	Study orientation and overview of the program structure PA foundations for children and families Parental support for child PA Confidence and perceived capability to support child PA Emotional influences on parental support behaviour Introduction to emotional regulation Introduction to ACT Values clarification around supporting child PA Parental identity reflection Committed action, goal setting, and support planning Take-home practice on emotions, values, commitment, and planning	**BCTO terms:** Inform about positive health consequences [BCIO:007183]; inform about positive emotional consequences [BCIO:007181]; instruct how to perform a behaviour [BCIO:007058]; affirm commitment [BCIO:007015]; identify self as role model [BCIO:007158]; affirm valued self-identity [BCIO:007159]; reframe past behaviour [BCIO:007056]; set behaviour goal [BCIO:007003]; action planning [BCIO:007010]; goal strategizing [BCIO:007008]. **ACT constructs:** values, self-as-context, committed action.	Primarily reflective, with clear reflexive identity content and regulatory planning.
2	Embracing the Moment to Support an Active Family	Review worksheet#1 and application of previous workshop material Acceptance-based responses to difficult parenting emotions Present-moment awareness and mindfulness skills Self-compassion in the context of parenting and support setbacks Defusion from unhelpful or self-critical thoughts Identifying emotional barriers to supporting child PA Applying ACT skills to real-life support challenges Take-home practice on mindfulness, acceptance, and defusion	**BCTO terms:** Self-monitor behaviour [BCIO:007024]; practise behaviour [BCIO:007094]; advise how to reduce negative emotions [BCIO:050344], including cognitive and behavioural strategies [BCIO:050337; BCIO:050333]; goal strategizing [BCIO:007008]. **ACT constructs:** acceptance, present-moment awareness, defusion.	Primarily regulatory, focused on emotional regulation and coping to protect enactment of parental support.
3	Skills and Strategies—Turning Intentions into Action	Review worksheet#2 and consolidation of previous workshop material Psychological flexibility in supporting child PA Self-monitoring of flexibility and follow-through Motivational self-regulation through positive self-talk Mental rehearsal and visualization of difficult support situations Implementation intentions and if-then planning Flexible responding through avoiding all-or-nothing thinking Persistence, resilience, and mental toughness for maintaining support behaviour Emphasis on consistency over perfection in family PA support	**BCTO terms:** Self-monitor behaviour [BCIO:007024]; prompt self-talk [BCIO:007140]; prompt mental rehearsal of successful performance [BCIO:007138]; action planning [BCIO:007010]; goal strategizing [BCIO:007008]. **ACT construct:** psychological flexibility.	Primarily regulatory, with maintenance-oriented support for repeated enactment and more stable follow-through over time.

Note. PA = physical activity; BCTO = Behaviour Change Technique Ontology; BCIO = Behaviour Change Intervention Ontology. For readability, the suffix “BCT” is omitted from individual BCTO labels in the table; all listed BCIO identifiers refer to BCTO behaviour change technique classes.

**Table 2:** Themes, Subthemes, and Selected Quotes Supporting the Themes.

Themes	Subthemes	Illustrative Quotes
Theme 1. Acceptable, Accessible, and Supportive Delivery	1a. Clear, Manageable, and Low-burden Delivery	“I found the workshops to be really efficient. I learned a lot. The presentation felt like it didn’t take a lot of my time or energy to do, as did the follow-up, and so the aesthetics kept me engaged with it, and it was a good presentation of material.”—Interview 08 “I thought they were well done. Some of the slides were a bit on the busy side, but maybe there could have been multiple slides. But I thought it was very, very well done. And it wasn’t too long. It was a good length, I thought… I enjoyed it. I learned a lot, so that was good.”—Interview 04 “I really enjoyed it, actually. It was really well laid out. I’m a visual learner, so it was really nice to be able to see all of that information as I was trying to process it.”—Interview 12
	1b. Supportive and Reusable Materials Enhanced Acceptability	“Because there was a couple of things that I wanted to kind of go back and look at again, so I appreciated being able to have the slides afterwards. That was helpful… it wasn’t too wordy. It also wasn’t dumbed down. It was very easy to understand and easy to apply to a family situation. The worksheets that came with it were also very helpful because they directly correlated… that wasn’t the case with this. It was very connected.”—Interview 11 “I really liked the presentations. They were really simple to understand and I liked that I have something to keep afterwards. I printed off a lot of the papers and laminated them… Some of them were just like infographics; those ones I just printed off, laminated, put them actually on my wall… The activities, those ones I actually modified a little bit to make them more general… and actually started using them with my son.”—Interview 09 “I wish I can take this one again to do a refreshment again. I do have the slides. It’s really useful. Really. Good knowledge.”—Interview 03
	1c. Validating and Relevant to Parenting Realities	“The actual meetings where we went over the PowerPoint presentations were very helpful, very insightful. It was that combination of support and education at the same time, that I felt validated by what was spoken about, but also what was in the slides that I was able to reflect on afterwards as well.”—Interview 11 “Just being easier on myself when things didn’t go that way, or if I wasn’t feeling I had the energy to do something. Knowing that’s okay… I try to be present for my kids, and in that process sometimes I’ll forget about the me piece. I think just being aware of how I’m feeling, or how that might affect being active with my kids, I think that was a good reminder, and something that I needed to hear.”—Interview 12 “It’s not so much the prescriptive, like, ‘You should do this, and you should do that.’ But more about that sort of underlying, like, ‘Okay, you know what to do now. You know that your kids need to be active. You know that you want to do it. It’s a priority for you. But how do you actually implement it?’ So I thought that was a kind of a different approach… and more supportive of the parents beyond just, ‘Here’s the recommendations.’”—Interview 04
Theme 2. Feasible Application with Perceived Impacts on Parental Support for Family PA	2a. Strategies were Generally Workable and Adaptable in Daily Family Life	“The biggest one for me and has had the biggest impact on getting my kids to be active is recognizing flexibility and letting go of the all-or-nothing thinking that I applied to myself. I can apply that to helping my kids be active… ‘Oh, it actually is quite hot out. So we’ll shorten our ride, or we’ll ride to the beach instead.’… Even if we get out for a 10 min walk after dinner, that’s better than nothing.”—Interview 08 “If every day it’s not possible to go outside, at least we will divide our work in between with our partner or parents. We can make an adjustment into our own schedule, but not to skip the play-date outside… maybe not both of us could be able to join for the day out, but one parent can go… we’ll try to figure out how we can make it possible for the child to go outside.”—Interview 01 “The all-or-nothing thinking, avoiding that, and just being in the moment… I think those were helpful, and probably the ones that I would more use first… I don’t really think there’s many barriers to implementing them. I think it’s easy once you get started.”—Interview 10
	2b. Parents Perceived Shifts in Mindset and Support Practices	“A lot of the same issues that came up for just myself, now they’re coming up for me with my son as well… the self-talk is the exact same. And I didn’t actually realize how much overlap there was until I started intentionally doing it… I only have half an hour before class to work out, so what’s even the point?… But obviously that’s not true… and the same thing with my son… half an hour is better than nothing at all.”—Interview 09 “I don’t think people realize the effect that their emotions, how much that affects the active lifestyle of their kids. It wasn’t something I considered until the study, so I don’t think I’m the only one.”—Interview 10 “I think that 3rd session focused more on how I manage myself and my perspective into inserting this into our lifestyle… All that mindfulness exercise helped reassure me that there is no perfect. There’s just making those baby steps and making an effort to do a little bit more on that end and not be too frustrated when I cannot do it right.”—Interview 05
	2c. Application was Shaped by Child Responsiveness and Family Complexity	“I think it is [feasible]. But again, I think me implementing it and getting results is sometimes a little bit difficult. You still need the other, the child to agree to it.”—Interview 06 “I think he or the study doesn’t fully grasp how complicated it can be in a household with multiple different dynamics, like neurodiversity or disabilities… I have 4 children… They are all neurodivergent… one loves nature, and one hates nature, and so trying to find that middle ground is and has been a little tricky.”—Interview 07 “When it’s just on me, I can, I feel that’s easier to do. But when I have to engage the kids, then there are more factors that come into play… my son has more work than he thought. My daughter is just not in the mode of actually just leaving the house… So that might be a way of making sure that not everything is just on me, as one person driving the initiative, but actually engaging the family as well.”—Interview 05
Theme 3. Recommendations for Refinement and Broader Fit	3a. More Support for Sustained Engagement	“The thing that I would really like is, the information is really good and you can read it, but it would be nice if those slides were made into little videos or something that you could just sort of refresh yourself with… then they can go back and they can review them whenever they want.”—Interview 04 “What it kind of lacks for someone as lazy as I am is that ongoing motivation. Some reminders would be very helpful to get us kickstarted as well… It might not be an email, maybe it’s just a notification from an app… to ensure the users stay engaged in this commitment that they have made.”—Interview 05 “If it was me, I think the sessions could be paced out a little bit more. Instead of checking in every week, maybe it should be 2 weeks… Let’s say if the session was every 2 weeks, and then the messages or the reminders could be maybe every 3 to 4 days… ‘Have you started?’ ‘How is week one going?’ ‘Do you need help?’”—Interview 05
	3b. More Interactive and Tailored Program Delivery	“If you and [first author] can provide like a 30 or 45 min in-person physical activities, then it could be good for the parents to not only visualize, but also do some actions together and some samples together to have a good trial in this program.”—Interview 02 “Where it didn’t work was, I think a lot of it was still based on the parent to figure out or the parent to do the work on it… what could have worked better in my situation was, if there was a workshop that more catered towards the child, and having him be more integrated into the study or into the workshop.”—Interview 06 “I think probably to do group sessions online would be really helpful. Then it would maybe allow for a little bit of a deeper dive… maybe going through some specific issues or examples with people… Six to ten parents maybe joining in at the same time.”—Interview 09

## Data Availability

The data presented in this study are not publicly available due to privacy and ethical restrictions related to qualitative interview transcripts. Requests to access de-identified data should be directed to the corresponding author and will be considered subject to ethics approval and consent restrictions.

## Appendix A

**Table A1:** Exit Interview Schedule.

These are the exit interview questions the researcher will verbally ask the parents after completing all workshops. **Workshop 1: Understanding Emotions to Support an Active Family** *“In the first workshop, we discussed the basics of physical activity, how emotions can influence your ability to support your child, and how values shape your parenting behaviors. With that in mind:”* **Workshop 2: Embracing the Moment to Support an Active Family** *“In the second workshop, we focused on strategies like acceptance, present-moment awareness, and self-compassion to help manage parenting stress. We also explored how to reduce the impact of unhelpful thoughts. Thinking about this workshop:”* **Workshop 3: Skills and Strategies—Turning Intentions into Action** *“In the final workshop, we talked about turning intentions into actions with tools like implementation intentions, avoiding all-or-nothing thinking, and using visualization and self-talk. Reflecting on this workshop:”* (1) How did you feel about the workshops? Please mention anything relating to the design of the slides, the information presented, the aesthetics of the presentation, or anything else you find relevant. (2) Do you feel there is demand for this study? Please comment on how relevant and in demand you felt about the material. (3) Is this program feasible to implement in your daily life? (4) Can you easily carry the workshop recommendations in your daily life? (5) Can you modify these lessons if unexpected events in your life come up quickly? (6) How easily can you integrate these lessons (for example, mindfulness and defusion) into your daily lives? (7) Can you use any of your previous experience with physical activities to apply to this study? (8) Do the ideas from this study, overall, fit within the dynamics of your family? (9) Did you find the material presented in the workshops new and innovative? Were there any concepts or strategies that you had not heard of before or found particularly insightful?
